# A metabolomics study in citrus provides insight into bioactive phenylpropanoid metabolism

**DOI:** 10.1093/hr/uhad267

**Published:** 2023-12-19

**Authors:** Shouchuang Wang, Shuangqian Shen, Chao Wang, Xia Wang, Chenkun Yang, Shen Zhou, Ran Zhang, Qianqian Zhou, Huiwen Yu, Hao Guo, Weikang Zheng, Xianqing Liu, Juan Xu, Xiuxin Deng, Qiang Xu, Jie Luo

**Affiliations:** Sanya Nanfan Research Institute, Hainan University, Sanya, 572025, China; Yazhouwan National Laboratory, Sanya 572025, China; Sanya Nanfan Research Institute, Hainan University, Sanya, 572025, China; Yazhouwan National Laboratory, Sanya 572025, China; Sanya Nanfan Research Institute, Hainan University, Sanya, 572025, China; National Key Laboratory for Germplasm Innovation & Utilization of Horticultural Crops, Huazhong Agricultural University, Wuhan 430070, China; Sanya Nanfan Research Institute, Hainan University, Sanya, 572025, China; Sanya Nanfan Research Institute, Hainan University, Sanya, 572025, China; Sanya Nanfan Research Institute, Hainan University, Sanya, 572025, China; National Key Laboratory of Crop Genetic Improvement and National Center of Plant Gene Research, Huazhong Agricultural University, Wuhan 430070, China; National Key Laboratory for Germplasm Innovation & Utilization of Horticultural Crops, Huazhong Agricultural University, Wuhan 430070, China; Sanya Nanfan Research Institute, Hainan University, Sanya, 572025, China; National Key Laboratory for Germplasm Innovation & Utilization of Horticultural Crops, Huazhong Agricultural University, Wuhan 430070, China; Sanya Nanfan Research Institute, Hainan University, Sanya, 572025, China; National Key Laboratory for Germplasm Innovation & Utilization of Horticultural Crops, Huazhong Agricultural University, Wuhan 430070, China; National Key Laboratory for Germplasm Innovation & Utilization of Horticultural Crops, Huazhong Agricultural University, Wuhan 430070, China; National Key Laboratory for Germplasm Innovation & Utilization of Horticultural Crops, Huazhong Agricultural University, Wuhan 430070, China; Sanya Nanfan Research Institute, Hainan University, Sanya, 572025, China; Yazhouwan National Laboratory, Sanya 572025, China

## Abstract

Citrus fruits are widely consumed worldwide in juices or as fresh and provide a broad range of phytonutrients that are important for human health. Here, a citrus multi-omics resource is presented: comprehensive metabolic profiling of various citrus species was performed and metabolic profiles were compared among species, with a focus on the phenylpropanoid metabolic pathway. A metabolite-based genome-wide association analysis (mGWAS) of 154 pummelo accessions was performed using factored spectrally transformed linear mixed models (FaST-LMM) and efficient mixed-model association eXpedited (EMMAX), and the genetic and biochemical basis of metabolomic variation was comprehensively analysed. A metabolite-single nucleotide polymorphism-gene (metabolite-SNP-gene) interaction network was constructed for pummelo, and many candidate loci controlling the synthesis and regulation of bioactive compounds were identified; among these loci, three BAHD malonyltransferases were involved in the malonylation of flavonoid glycosides. Further investigation revealed that an R2R3-MYB transcription factor CgMYB1 positively controls the metabolism of phenylpropanoid molecules, particularly the flavonoid derivatives. This study provides valuable data resources on the metabolic regulatory networks of bioactive components in citrus, in addition to demonstrating an efficient method for metabolic pathway dissection and providing targets for future breeding work with the aim of improving nutritional value.

## Introduction

Citrus species are widely consumed worldwide in juices or as fresh fruit. Citrus fruits, as a type of ‘sink’, accumulate high levels of secondary metabolites, such as carotenoids, flavonoids, and terpenoids, as compared to both other tissue types and fruits from other species. Fruit flavor is determined by species identity and the levels of various metabolites, which also provide a wide range of phytochemicals important for human nutrition [1]. In 2012, the global citrus acreage was nine million hectares, with a total output of more than 86 million metric tons and an estimated value of US$9 billion (United Nations Food and Agriculture Organization) [2]. Citrus fruit trees (Citrinae) represent a large group belonging to *Aurantioideae* and *Rutaceae*. It is believed that citrus species originated in Southeast Asia and were domesticated about 4000 years ago. The Citrinae is divided into three groups of species: primitive citrus, near citrus, and true citrus. True citrus includes all commercially cultivated citrus, including *Citrus limon* (lemon), *Citrus maxima* (pummelo), *Citrus paradisi* (grapefruit), *Citrus reticulata* (tangerine and mandarin), and *Citrus sinensis* (sweet orange) [3]. Among cultivated species, pummelo currently has the most genetic data available [4, 5]. Citrus species synthesize and accumulate a wide variety of phytochemicals, including flavonoids, hydroxybenzoic acid, hydroxycinnamic acid, stilbene, terpenoids, and other low-molecular weight phenols. Pummelo not only contains these nutrients, but is also rich in antioxidants, which can protect cells from free radicals and lower blood pressure and cholesterol [6]. Characterizing the pummelo metabolome, its variation among cultivars, and its genetic basis will promote the improvement of citrus nutritional quality and the cultivation of new varieties.

Plant phenylpropanes are a class of secondary metabolites with physiological activity produced by phenylalanine, including coumarins, flavonoids, phenolic acids, etc. They are not only bioactive ingredients involved in plant development and defense [7–9], but also have many health benefits, such as anti-cancer and anti-inflammatory properties [10]. Citrus species are notable for containing large quantities of phenylpropanoids, especially flavonoids and phenolic acids, and are one of the major sources of phenlypropanoids in the human diet, as well as a primary source for the food and para-pharmaceutical industries. Flavonoids represent a major branch of the phenylpropane pathway and one of the largest families of plant secondary metabolites [11]. Flavonoids are derived from two precursors: phenylalanine and malonyl coenzyme A. The two phenyl rings (A and B) are connected by a pyran ring (C), which together constitute the basic flavonoid structure. Different kinds of modifications occur at different positions on the diphenyl propane backbone (C6-C3-C6), including acylation, glycosylation, hydroxylation, and methylation [8, 12]. A variety of flavonoids have been identified from citrus species; for example, hesperidin in orange and naringin in pummelo. The fruit cortex of some species also contains a specific class of flavonoids, polymethoxyflavones, which are almost exclusively present in citrus plants [13, 14]. The flavonoids that accumulate in citrus fruits predominantly include flavanone-*O*-glycosides and flavanone-*O*/*C*-glycosides and their derivatives; flavanone 7-*O*-glycosides are the most abundant flavonoid type in all citrus fruits [15]. Citrus is a major source of flavonoids in the human diet; when consumed, flavonoids are bioactive with significant health benefits. Citrus flavonoids have greater antioxidant properties than ascorbic acid and are therefore used in anticancer studies [16]. For example, naringin can inhibit prostate cancer cells by inducing DNA repair [17]. Similarly, citrus polymethoxyflavonoids show strong anticancer activity, inhibiting the proliferation of a variety of cancer cells, including from lung cancer and melanomas, as well as inhibiting the metastasis of gastric cancer tumor cells; thus, polymethoxyflavonoids have high potential as anticancer drugs [18]. Citrus flavonoids also have superior antibacterial, anti-inflammatory, and blood glucose lowering effects, and hesperidin, naringin, and polymethoxyflavonoids may help combat cardiovascular disease and obesity [19]. Montbretin A, a complex acylated flavonol glycoside, can efficiently and specifically inhibit human pancreatic amylase (HPA) activity, thus effectively reducing blood glucose levels [20]. In addition to their human health benefits, flavonoids also play an important role in the growth and flavor of citrus plants/fruits themselves; for example, the anthocyanins synthesized by most citrus cultivars determine fruit color [21, 22], and naringin is one of the three main bitter compounds in citrus [23]. Although many studies have examined citrus flavonoid metabolic pathways, the biosynthetic networks of complex flavonoid derivatives remain to be explored.

Metabolites are the end products of cellular processes, and their abundance can be considered as indicators of genetic or environmental changes in biological systems. A widely targeted metabolomics method with high sensitivity and coverage has recently been developed [24] and has been applied in major crops such as citrus [25, 26], maize [27], rice [28, 29], tomato [30], and others. More systematic research on citrus, especially in pummelo, will promote the improvement of citrus nutritional quality and provide new resources for citrus breeding. Genome-wide association studies (GWAS) were initially utilized in the model species *Arabidopsis thaliana* [31], before being applied in a number of other plant species, including major crops [28, 30]. However, there have been relatively few recent advances in metabolomics research in citrus, and there are no reported case studies using natural populations and mGWAS to analyse the genetic and/or biochemical basis of metabolome variation in pummelo. Therefore, more research is needed to identify intermediate traits related to the biochemistry and physiology of citrus species.

Here, a comprehensive metabolic analysis was performed for various citrus species and metabolic differences among species were compared, with a focus on the phenylpropanoid metabolic pathway. A metabolite-based genome-wide association study (mGWAS) of 154 pummelo accessions was carried out to determine the genetic and biochemical basis of metabolome variation. Numerous high-confidence candidate genes were associated with metabolite content; candidate genes involved in the biosynthesis of flavonoid derivatives were validated in detail. Further analysis showed that a R2R3-MYB transcription factor, CgMYB1, positively regulates phenylpropanoid metabolism. Together, the interaction network constructed in this study (based on metabolite diversity) and the mGWAS results provide valuable data resources for further research on the biosynthetic pathways and regulatory mechanisms of important natural products in citrus.

## Results

### Metabolic profiling of citrus varieties

In order to investigate the differences of metabolic profiles of citrus varieties, Chinese citrus varieties with different geographical origin, consumption type, and improvement status were collected in this study. This collection comprises a total of 189 citrus accessions (Table S1, see online supplementary material), including 154 pummelo and 35 other citrus accessions. A widely targeted MS2 spectral tag (MS^2^T) library was constructed using liquid chromatography–tandem mass spectrometry (LC–MS/MS); the final library contained 994 high-quality (s/n > 10) metabolic signals complete with product ion spectra (MS^2^) and retention time (RT) (Table S2, see online supplementary material). The structures of 360 metabolites were identified by comparison of mass spectral fragments and retention times of standards, manual annotation of mass spectral profiles and searching of metabolic databases; these included primary metabolites of amino acids and vitamins, as well as secondary metabolites such as lipids and phenylpropanoids. Different types of citrus metabolome databases were constructed, and a semi-quantitative analysis was performed via liquid chromatography-mass spectrometry (LC–MS) (Table S3, see online supplementary material).

**Figure 1:**
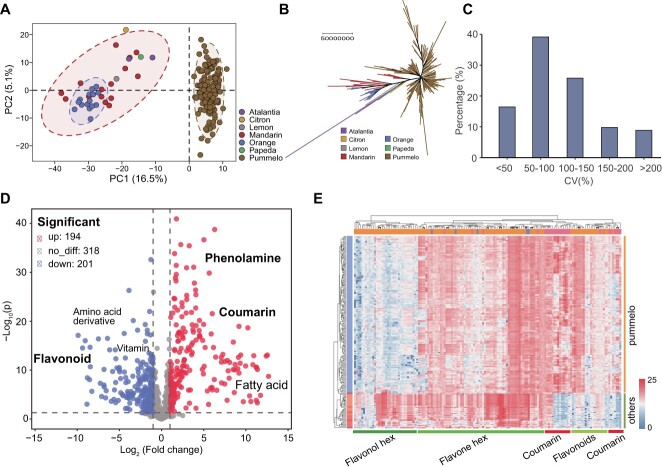
Comparison of metabolic profiles among citrus varieties. **A** PCA analysis of metabolites identified in the fruit tissues of various citrus species. A total of 189 different types of citrus germplasm resources were used in the PCA, including 154 pummelos, as well as 15 madarin, 15 oranges, one lemon, one citron, one papeda, and two *Atalantia buxifolia*. **B** Neighbor-joining phylogenetic tree constructed using metabolites identified in different citrus species. The scale bar indicates the simple matching distance. **C** Histogram of coefficient of variation values for relative metabolite content in the pummelo accessions. **D** Volcano plot of the differences in metabolite content between pummelo and other citrus species, including sweet orange and mandarins. The red dots and blue dots represent metabolites that have increased or decreased in content, respectively, with a fold change ≥2.0 or ≤ 0.5 and *P-*value ≤0.05. **E** Heatmap of relative differences in metabolite content in pummelo versus other citrus fruits. The content of each metabolite was normalized before performing hierarchical clustering. Yellow indicates high metabolite abundance, and blue indicates low abundance. Abbreviations: hex, hexoside.

To obtain an overview of metabolic variation in individual *Citrus* species, the metabolites of citrus cultivars were analysed by principal component analysis (PCA). In the PCA plot, the pummelo accessions were separated from other citrus species (two distinct clusters), reflecting the uniqueness of the pummelo metabolic profile (Fig. 1A; Table S4, see online supplementary material). Of the known metabolites, the main contributors to interspecific variation were coumarin and the flavonoids, suggesting that differences in metabolism among citrus varieties primarily occur in the phenylpropanoid biosynthesis pathway (Table S4, see online supplementary material). Similarly, a neighbor-joining tree of the citrus metabolome also distinguished pummelo from other citrus species, and the pummelo accessions were further divided into two subgroups (Fig. 1B). The topography of the metabolite-based evolutionary trees was also consistent with previously reported genetic relationships among citrus species [5]. Across citrus species, 92.8% of the metabolic features showed coefficients of variation (CVs) greater than 0.5, of these, 86% (855 of 994) occurred in the pummelo population. Thus, most metabolic features had significant variation, rendering the data suitable for GWAS (Fig. 1C). Comparative metabolic profiling was used to identify metabolites varying significantly in abundance among citrus species; these included amino acids (and their derivatives), coumarins, fatty acids, flavonoids, phenolamines, and vitamins (Table S3, see online supplementary material). Compared to mandarins and sweet oranges, there were 194 significantly up-regulated and 201 significantly down-regulated metabolites in pummelo (Fig. 1D; Table S5, see online supplementary material). A clustering analysis revealed variation in the abundance of different coumarin and flavonoid structural types across the 189 natural citrus populations, with flavonoids being the most abundant metabolite. The flavonoid content was lower in pummelo than in other citrus species, while the coumarin content was generally higher (Fig. 1E).

### Comparative analysis of multiple mGWAS in pummelo populations

Broad-sense heritability (*H^2^*) was calculated for all detected metabolites to determine the genetic basis. Most metabolites (878/994, or 88.3%) had *H^2^* values higher than 0.5, and 495 (49.8%) metabolites had *H^2^* values higher than 0.7 (Fig. S1, see online supplementary material). In addition to the high *H^2^* detected for secondary metabolites such as coumarins and flavonoids, relatively high *H^2^* values were also observed for some primary metabolites (Table S2, see online supplementary material).

**Figure 2:**
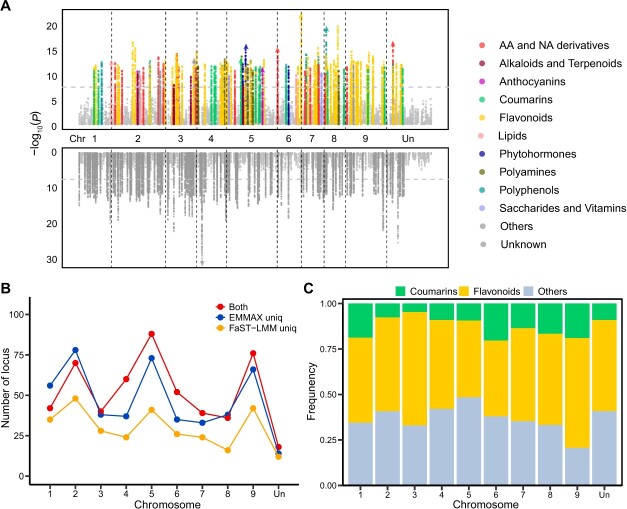
mGWAS results and analysis from FaST-LMM and EMMAX. **A** Manhattan plots of mGWAS results with top loci (10%) illustrated for each metabolite class. Different colors represent different classes of metabolites. **B** The number of unique and common outlier loci obtained by the two mGWAS programs plotted by chromosome. Blue, orange, and red represent unique loci from FaST-LMM, unique loci from EMMAX, and common outlier loci, respectively. **C** The proportion of coumarin- and flavonoid-associated loci by chromosome.

A total of 1 970 314 SNPs were identified from the genome sequencing data for the 154 pummelo accessions using the reference pummelo genome (database available from citrus.hzau.edu.cn). Of these, 103 720 nonsynonymous SNPs were distributed in 27 196 genes. After imputation, 1 895 975 common SNPs (minor allele frequency >0.05 and missing ratio <10%) were available for mGWAS, which was carried out in FaST-LMM and EMMAX. A Bonferroni-corrected *P*-value of 4.17e^−8^ was used as the genome-wide significance threshold for all traits (Table S6, see online supplementary material). A total of 11 932 repeated lead SNPs (7279 unique lead SNPs) were detected for different metabolites; of these SNPs, 2062 were only detected by FaST-LMM (Table S7, see online supplementary material), 4703 were only detected by EMMAX (Table S8, see online supplementary material), and 5167 were detected by both programs (Table S9, see online supplementary material). As shown in Fig. S2 (see online supplementary material), 79.7% of metabolites (792 out of 994 metabolites) had at least one significant association, with an average of 3.49 associations per metabolite. Considering the false-positive and false-negative issues associated with GWAS, Tables S11 andS12 (see online supplementary material) provide a full list of significant and highly significant associations that can be useful for further candidates exploration and other studies. After merging all results, the number of metabolite-associated loci increased (Fig. S3, see online supplementary material). A total of 2761 outlier loci were detected in at least one program (i.e., EMMAX or FaST-LMM), of which 1093 loci were detected more than once (Table S10, see online supplementary material). Manhattan plots of the significant loci illustrate outlier sites for each metabolite class, including top loci (134 of all 1285 loci) for amino acids (and their derivatives), coumarins, flavonoids, nucleic acids (and their derivatives), phenolamides, terpenoids, and other known metabolites (Fig. 2A). Across all known metabolites, FaST-LMM and EMMAX identified 296 and 468 unique loci, respectively. These were mainly distributed on chromosomes 2, 5, and 9 (Fig. 2B). In addition, flavonoid and coumarin loci accounted for a large proportion of all outlier loci on each chromosome, from 41.59%–62.26% and 4.72%–20.35%, respectively (Fig. 2C). This suggests an extensive genetic regulatory network for these two phenylpropane metabolic derivatives.

### Metabolite-SNP-gene networks in the pummelo metabolome

**Figure 3:**
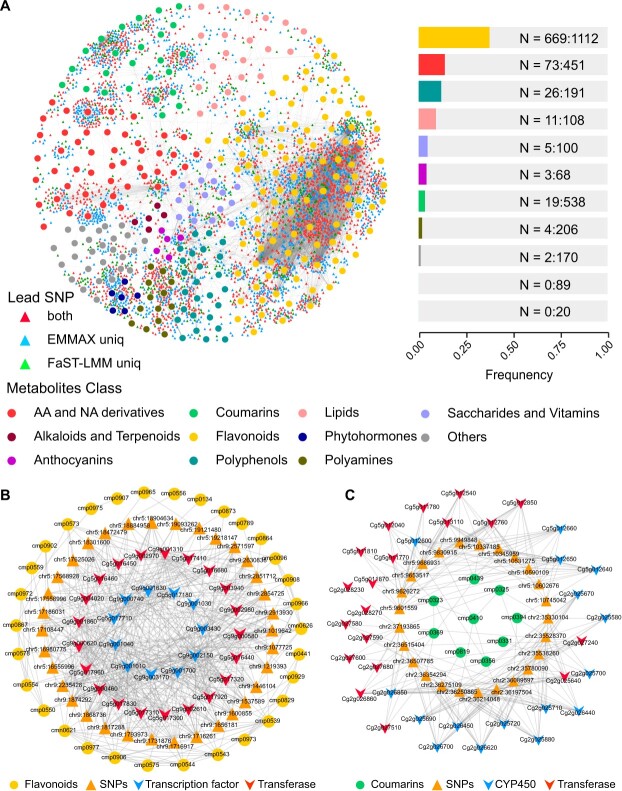
Associations between metabolite content and lead SNPs. **A** Network diagram (on the left) illustrating associations between metabolites and lead SNPs. Triangles of different colors represent different types of lead SNPs (red for common, blue for EMMAX program-specific, and green for FaST-LMM program-specific), while circles of different colors represent different classes of metabolites. Bar plot (on the right) illustrating the number of lead SNPs associated with multiple metabolites (Multiple) versus a single metabolite (Single) for each class of metabolites, where N = Multiple: Single. **B** Expanded flavonoid subnetwork diagram. Triangles represent lead SNPs, circles represent metabolites, and inverted triangular prisms represent genes. **C** Expanded coumarin subnetwork diagram. Triangles represent lead SNPs, circles represent metabolites, and inverted triangular prisms represent genes.

To further explore the relationships between metabolite content and outlier SNPs, an association analysis based on 295 metabolites and 3713 lead SNPs was used to construct a metabolite-SNP interaction network. The interaction network contained a total of 5906 significant associations (between various metabolites and lead SNPs), with the most complex associations observed for flavonoid content (Fig. 3A). A total of 669 SNPs were associated with multiple flavonoids, and 1112 SNPs were associated with a single flavonoid. Other metabolites, such as amino acids and their derivatives, coumarins, and lipids, also showed metabolite-SNP associations (Fig. 3A).

A metabolite-SNP-gene interaction network was constructed including diverse modifying groups for each metabolite types and metabolic reaction types, as well as genetic loci represented by lead SNPs; the network was used to screen candidate genes for metabolic synthesis and regulation. The flavonoid subnetwork contained 34 lead SNPs, 31 flavonoids, 21 transferases, and 11 transcription factors (Fig. 3B, Table S13, see online supplementary material). All lead SNPs were associated with at least four flavonoids. Multiple methyltransferases (e.g., *Cg9g000460* and *Cg9g001860*), malonyltransferases (*Cg5g017300*, *Cg5g017310*, and *Cg5g017830*), and an acetyltransferase (*Cg9g001310*) were associated with the lead SNPs (Table S14, see online supplementary material), as well as two bHLHs (*Cg5g017710* and *Cg9g001040*), two MYBs (*Cg9g000740* and *Cg9g001630*), and a WRKY transcription factor (*Cg9g001010*) (Table S14, see online supplementary material). Similarly, the coumarin subnetwork contained 25 lead SNPs, nine coumarins, 19 transferases, and 16 cytochrome P450s (Fig. 3C, see online supplementary material). The transferases included multiple UDP-glycosyltransferases (e.g., *Cg2g027580*, *Cg2g027590*, and *Cg2g027600*) (Table S14, see online supplementary material).

### Functional characterization of candidate genes underlying mGWAS associations

**Figure 4:**
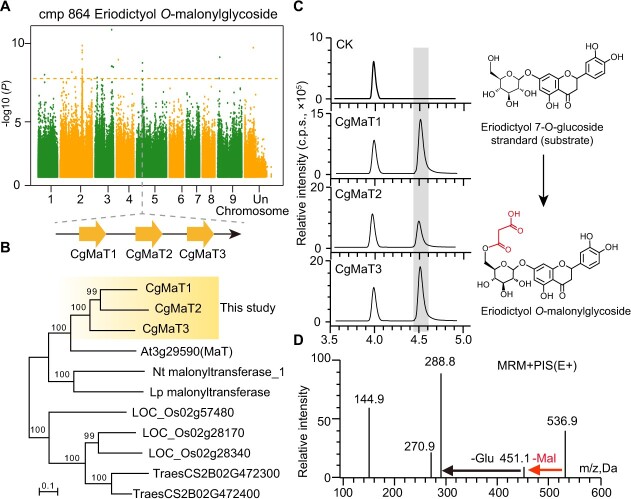
Identification and functional analysis of CgMaTs. **A** Manhattan plot displaying the mGWAS results for metabolite cmp864 (eriodictyol *O*-malonylglycoside). Association strength is illustrated as the negative logarithm of the *P*-value from the linear mixed model. Three adjacent acyltransferase-coding genes (i.e., *Cg5g017300*, *Cg5g017310*, and *Cg5g017830*) are designated as candidates. **B** Phylogenetic analysis of the three CgMaTs. Bootstrap values >70% (based on 1000 replicates) are indicated at each node (scale bar: 0.1 amino acid substitutions per site). **C** HPLC chromatograms illustrating the reaction of each CgMaT with malonyl-CoA and eriodictyol 7-*O*-glucoside as the substrate. **D** The MS spectrum and chemical structure of the product eriodictyol *O*-malonylglycoside generated from the enzymatic assays.

Flavonoid metabolic diversity is largely due to chemical modifications of the basic skeleton, such as acyl modifications [32]. To further characterize the flavonoid modification network in pummelo, cmp864 (eriodictyol *O*-malonylglycoside) was taken as an example to locate and verify underlying biosynthetic genes. Three duplicate genes annotated as acyltransferases (*Cg5g017300*, *Cg5g017310*, and *Cg5g017830*) were identified as candidate genes responsible for the acyl modification step (Fig. 4A, Table 1). Sequence alignment revealed an ortholog in *A. thaliana*, *At3g29590* (Fig. 4B), that encodes a malonyl-CoA: anthocyanidin 5-*O*-glucoside-6”-*O*-malonyltransferase (MaT). Thus, the three candidate genes were named *CgMaT1*, *CgMaT2*, and *CgMaT3*. To characterize these candidate genes, the open reading frame of the candidate acyltransferase gene was amplified from *Citrus grandis* ‘*Wanbaiyou*’ and cloned and heterologous expressed in *Escherichia coli*, then purified for enzymatic analysis. The result shows that all three conferred malonyl-transferase activity on eriodictyol *O*-glycoside (Fig. 4C and D). Considering that the BAHD gene family is characterized by substrate promiscuity, two other flavonoids, flavone glucoside (luteolin 7-*O*-glucoside) and flavonol glucoside (quercetin 5-*O*-glucoside), were also used for enzyme activity tests, due to their structural similarity. Both flavonoids conferred malonyl-transferase activity on luteolin 7-*O*-glucoside and quercetin 5-*O*-glucoside, while varying in their relative enzyme activity (Fig. S4, see online supplementary material). Overall, these results show that our program to locate key metabolic pathway genes is effective and efficient.

**Table 1:** Summary of candidate genes for flavonoid and coumarin association in mGWAs results.

**Metabolite**	**Classes**	**Lead SNP**	**Candidate gene**	**Description**	**Distance (Mb)**	**Method**	**Function identification/annotation**
Kaempferol-3-*O*-rhamnosylhexoside-7-*O*-rhamnoside	Flavonoid	chr1:1742935	Cg1g002060	Flavanone 3-hydroxylase	0.44	Both	
Kaempferol-3-*O*-rhamnosylhexoside-7-*O*-rhamnoside	Flavonoid	chr1:7022888	Cg1g009050	Flavonol synthase	1.02	EMMAX	[54]
Kaempferol-3-*O*-rhamnosylhexoside-7-*O*-rhamnoside	Flavonoid	chr7:5714861	Cg7g006780	Phenylalanine ammonia lyase	0.12	EMMAX	
Phellopterin	Coumarin	chr1:13500188	Cg1g016490	Dehydrogenase	0.11	Both	
Phellopterin	Coumarin	chr1:17066735	Cg1g019820	Prenyltransferase	0.19	Both	
Phellopterin	Coumarin	chr1:20843015	Cg1g022010	Beta-amyrin synthase	0.08	Both	
Poncirin	Flavonoid	chr2:8568031	Cg2g009540	Chalcone synthase	0.05	EMMAX	[55]
Eriodictyol *O*-malonylglycoside	Flavonoid	chr5:9786739	Cg5g017300	Transferase	7.73	EMMAX	In vitro
Eriodictyol *O*-malonylglycoside	Flavonoid	chr5:9786739	Cg5g017310	Transferase	7.75	EMMAX	In vitro
Eriodictyol *O*-malonylglycoside	Flavonoid	chr5:9786739	Cg5g017830	Transferase	8.64	EMMAX	In vitro
Luteolin 7-*O*-glucoside	Flavonoid	chr5:17186031	Cg5g017300	Transferase	0.33	Both	In vitro
Luteolin 7-*O*-glucoside	Flavonoid	chr5:17186031	Cg5g017310	Transferase	0.35	Both	In vitro
Luteolin 7-*O*-glucoside	Flavonoid	chr5:18301600	Cg5g017830	Transferase	0.13	Both	In vitro
Bergamottin	Coumarin	chr2:36354294	Cg2g026620	Cytochrome P450	0.06	EMMAX	
Kaempferol 3-*O*-glucoside-2'-*O*-rhamnoside	Flavonoid	chr3:28501573	Cg3g023030	Dihydroflavonol-4-reductase	0.05	Both	
Kaempferol 3-*O*-glucoside-2'-*O*-rhamnoside	Flavonoid	chr9:1874292	Cg9g001630	Myb transcription factor	0.06	Both	In vivo
Luteolin	Flavonoid	chr5:8225622	Cg5g011190	Flavonoid 3′-hydroxylase	0.01	Both	
Bergapten	Coumarin	chr4:1468804	Cg4g001710	Prenyltransferase	0.39	EMMAX	
Didymin	Flavonoid	chr4:25671290	Cg4g020580	Cytochrome P450	0.06	Both	
Limocitrin O-hexoside	Flavonoid	chr5:41668023	Cg5g034000	Myb transcription factor	0.15	Both	
Hydroxy-bergamottin	Coumarin	chr5:9686931	Cg5g012760	Prenyltransferase	0.41	Both	
Dihydrokaempferol-7-*O*-rutinoside	Flavonoid	chr5:28306932	Cg5g022560	Myb transcription factor	0.25	FaST-LMM	[44]
Apigenin-6-*C*-glucoside-*O*-pentoside	Flavonoid	chr6:23514828	Cg6g025740	Glycosyltransferase	0	EMMAX	[56]
Apigenin-6-*C*-glucoside-*O*-pentoside	Flavonoid	chr6:23514828	Cg6g025760	Glycosyltransferase	0	EMMAX	
Apigenin-6-*C*-glucoside-*O*-pentoside	Flavonoid	chr6:23514828	Cg6g025770	Glycosyltransferase	0	EMMAX	
Apigenin-6-*C*-glucoside-*O*-pentoside	Flavonoid	chr6:23514828	Cg6g025780	Glycosyltransferase	0	EMMAX	
Melitidin	Flavonoid	chr2:23018791	Cg2g016620	Acyltransferases	1.04	Both	[45]
Melitidin	Flavonoid	chr9:36821508	Cg9g025790	Acyltransferases	0	FaST-LMM	[45]
Melitidin	Flavonoid	chr9:36821508	Cg9g025800	Acyltransferases	0.04	FaST-LMM	[45]
Melitidin	Flavonoid	chr1:18083172	Cg1g023820	Rhamnosyltransferase	6.68	Both	[45]
Naringenin 7-*O*-glucoside	Flavonoid	chr9:38284124	Cg9g027850	Glycosyltransferase	0.05	EMMAX	[45]
Naringenin 7-*O*-glucoside	Flavonoid	chr9:38284124	Cg9g027920	Glycosyltransferase	0.08	EMMAX	[45]
Naringenin 7-*O*-glucoside	Flavonoid	chr9:38284124	Cg9g027930	Glycosyltransferase	0.09	EMMAX	[45]
Naringenin 7-*O*-glucoside	Flavonoid	chr9:38284124	Cg9g027970	Glycosyltransferase	0.12	EMMAX	[45]
Neohesperidin	Flavonoid	chr3:2312309	Cg3g000970	Glycosyltransferase	0.7	EMMAX	
Neohesperidin	Flavonoid	chr3:2312309	Cg3g000980	Glycosyltransferase	0.69	EMMAX	
Neohesperidin	Flavonoid	chr3:2312309	Cg3g001140	Glycosyltransferase	0.47	EMMAX	
Neohesperidin	Flavonoid	chr7:21945307	Cg7g022600	Glycosyltransferase	0.46	EMMAX	

### CgMYB1 positive regulates phenylpropanoid metabolism in pummelo

**Figure 5:**
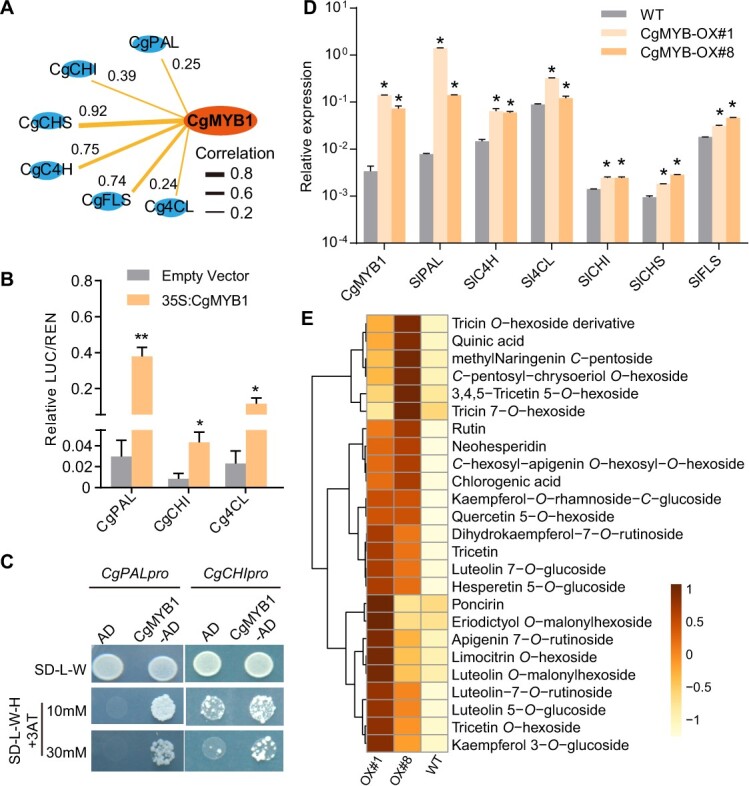
CgMYB1 positively controls phenylpropanoid metabolism. **A** Correlation network for structural genes of the phenylpropanoid biosynthesis pathway and *CgMYB1*. Pearson correlation coefficient values were calculated for each pair of genes. Genes in phenylpropanoid biosynthesis are shown as follows: *PAL*, phenylalanine ammonia lyase; *CHI*, chalcone isomerase; *CHS*, chalcone synthase; *C4H*, cinnamate 4-hydroxylase; *FLS*, flavonol synthase; *4CL*, 4-coumarate CoA ligase. **B** A dual luciferase reporter assay in *Nicotiana benthamiana* revealed that CgMYB1 can activate the promoters of the tested genes. LUC activity was normalized to REN activity as an internal control. Error bars indicate standard deviations (SDs) from three replicates. Asterisks indicate significant differences as determined by *t*-tests (*P* < 0.05). **C** Binding tests for CgMYB1 to the promoters of tested genes in a yeast one-hybrid assay. Each of the promoters was fused to a His2 reporter, and CgMYB1 was fused to GAL4 AD. **D** Relative expression analysis of endogenous phenylpropanoid biosynthesis genes in CgMYB1-overexpressed tomato lines based on quantitative RT-PCR. Data are means ±SDs of two independent biological replicates. Asterisks (*P* < 0.05, Student’s *t*-tests) indicate significant differences compared with the WT (wild type). **E** The flavonoid content in leaves from CgMYB1-OX transgenic tomato plants. The data are presented as means ±SDs, *n* = 3. Expression data were Z-score standardized.

Flavonoids represent the main pathway of phenylpropanoid metabolism. It has been reported that phenylpropanoid pathway genes are predominantly regulated by the R2R3-MYB transcription factors [33, 34]. A putative flavonol-related MYB transcription factor (named CgMYB1, *Cg9g001630*) was identified from the genome-wide analysis of the pummelo genome and a phylogenetic analysis (Table 1; , see online supplementary materialFig. S5). The subcellular distribution of CgMYB1 was analysed by transient expression in tobacco leaves, results showed that the green fluorescence produced by CgMYB1-GFP overlated with the red fluorescence produced by *OsGhd7*-RFP (nuclear positive control), suggesting that CgMYB1 is a nuclear protein and may function as a transcription factor (Fig. S5, see online supplementary material). Further expression profiling of phenylpropanoid metabolism genes and the candidate transcription factor showed that *CgCHI*, *CgCHS*, *CgPAL*, *CgFLS,* and *Cg4CL* were strongly co-expressed with CgMYB1 (Fig. 5A), suggesting that CgMYB1 could potentially moderate phenylpropanoid metabolic pathways, in particular the flavonoid biosynthesis pathways.

To further investigate whether CgMYB1 directly induces the expression of phenylpropanoid metabolism genes, dual luciferase reporter assays were performed in tobacco. As a first step, the promoters of the biosynthetic genes involved in phenylpropanoid metabolism (*CgCHI*, *Cg4CL*, and *CgPAL*) were cloned. Promoter activity for all biosynthetic genes was significantly higher when CgMYB1 was expressed than in the control (Fig. 5B), indicating that CgMYB1 is a positive regulator of phenylpropanoid metabolism. Furthermore, a yeast-one-hybrid assay indicated that CgMYB1 binds to the *CgCHI*, and *CgPAL* promoters (Fig. 5C). Thus, CgMYB1 regulates flavonoid biosynthesis by modulating the transcript levels of *CgCHI* and *CgPAL*.

To investigate the potential metabolic function of *CgMYB1* in plants, *CgMYB1* was transiently overexpressed in tobacco and the content of flavonoid was greater in all overexpression lines (Fig. S5, see online supplementary material). Next, another dicotyledonous plant, tomato, was used to construct *CgMYB1* overexpression lines. A qRT–PCR analysis of the CgMYB1-OX transgenic lines and WT samples was performed; the expression of flavonoid biosynthetic genes, including *Sl4CL*, *SlC4H*, *SlCHI*, *CgCHS*, *CgPAL*, and *CgFLS*, was significantly higher in the transgenic lines (Fig. 5D). Furthermore, metabolic profiling performed using LC–MS indicated that specific phenylpropanoid compounds (e.g., eriodictyol *O*-malonylhexoside, luteolin *O*-malonylhexoside, quercetin 5-*O*-hexoside, and tricin 7-*O*-hexoside) were markedly altered in abundance by *CgMYB1* overexpression. Additionally, their corresponding derivatives also showed enhanced abundance in these transgenic lines (Fig. 5E). The above results suggest an essential role for CgMYB1 in positively regulating flavonoid biosynthesis in pummelo.

## Discussion

Plants represent a vast storehouse of natural products from which new or potentially useful metabolites, such as medicines and nutrients, can be found. In contemporary metabolomics, important research topics include documenting plant secondary metabolites, analysing their synthesis and regulation mechanism, and to elucidate the diversity and natural variation of metabolites in the evolutionary process is one of the hot topics in the field of botany research [35]. Citrus production and acreage rank first among all fruit crops. When immature, citrus fruits are also used as raw materials for traditional folk medicine, despite their bitter taste, which is conferred by high levels of flavonoids. Some pummelo landraces are also used in folk medicine. Pummelo plants undergo typical sexual reproduction, in contrast to most other citrus species in which reproduction occurs via apomixis; pummelo is thus more genetically diverse than other citrus species (Fig. 1). A systematic analysis of the pummelo metabolome and underlying genes will greatly support improvement efforts for citrus nutritional quality. Analysing the nutritional components of citrus and uncovering the genetic basis of their synthesis and regulation is an important prerequisite for the development of improved citrus varieties. Such a study would also provide genetic resources and a theoretical basis for the development of new varieties with improved nutritional quality.

As sequencing technologies have advanced, integrative approaches such as genomics, transcriptomics, and metabolomics now play increasingly important roles in analyzing variation in metabolites, mining structural genes and transcription factors, and characterizing entire metabolic pathways [36, 37]. The metabolic and genetic variation in natural pummelo populations provides a material resource for the analysis of pummelo metabolic pathways and their molecular underpinnings (Fig. 2). Using natural pummelo populations, this study used a genome-wide association analysis to efficiently identify key genes controlling metabolite synthesis and regulation, including genes involved in eriodictyol *O*-malonylglycoside biosynthesis (Fig. 4, Table 1). Flavonoid glycosides, which are comprised of a sugar unit bound to a phenol aglycone, are among the most abundant secondary metabolites in citrus. In plants, malonylation represents an important modification of phenolic glycosides that is regulated by flavonoid glucoside malonyltransferases. In this study, three candidate genes (*CgMaTs*) encoding flavonoid glucoside malonyltransferases were identified using mGWAS and a subsequent evolutionary analysis (Fig. 4A). Further *in vitro* enzyme activity assays confirmed the activity of the gene products, furthering our understanding of the malonyl-modified flavonoside metabolic network in pummelo (Fig. 4B). Notably, the BAHD family enzymes responsible for flavonoid glycosylation usually have relatively poor substrate specificity. Here, the CgMaTs conferred malonyl-transferase activity on different flavonoid glucosides, including flavanone glucoside (eriodictyol *O*-glycoside), flavone glucoside (luteolin 7-*O*-glucoside), and flavonol glucosides (quercetin 5-*O*-glucoside) (Fig. S4, see online supplementary material), confirming the generality of malonyltransferases. Similarly, recent studies in wheat and rice have identified key genes for flavonoid glucoside malonyltransferases [28, 38], indicating the high efficiency of the mGWAS approach for gene mapping. Furthermore, malonylation of flavonoid glycosides plays an important role in various processes, as previously described [39, 40]. Excess flavonoid glycosides are detrimental to plant growth and development, and acylation (catalyzed by malonyltransferases) is an important way to consume flavonoid glycosides to reduce these detrimental effects. A recent study demonstrated the molecular mechanisms underlying the antitoxic effect of BtPMaT1 on flavonoid glycosides in whitefly, providing a basis for the development of target gene-directed, environmentally friendly control technology for whitefly [41]. Additionally, in *A. thaliana*, BAHD malonyltransferase activity influences anthocyanin stability at neutral pHs, and acylation may also affect the anthocyanin absorption maxima [39]. Whether malonylation of flavonoid glycosides results in similar effects on pummelo physiology remains to be determined. Taken together, these findings suggest that further flavonoid pathway dissection is of key importance for understanding nutritional content, as well as other complex physiological traits, in pummelo.

Citrus plants contain a large number of flavonoids and represent the main flavonoid source in human diets [42, 43]. Flavonoids not only play an important role in citrus plant growth and development, but also determine plant nutritional and medicinal qualities [26]. Therefore, characterizing the regulatory networks moderating flavonoid content will permit better utilization of citrus metabolite resources in the future [44, 45]. To date, several transcription factors regulating important metabolic processes have been identified in citrus plants; for example, the MYB family transcription factors CgRuby1 and CgRuby2 regulate anthocyanin biosynthesis [22]. Meanwhile, a R2R3-MYB transcription factor CsMYBF1 has been discovered to regulate flavonoid synthesis in sweet orange [46]. Here, the role of the R2R3-MYB transcription factor CgMYB1 was functionally verified in pummelo. Yeast-one-hybrid and dual luciferase assays demonstrated that CgMYB1 directly binds to the promoters of *CgCHI*, and *CgPAL*, activating transcription (Fig. 5B and C). *CgPAL* is an upstream gene in the phenylpropane pathway, and *CgPAL* activation may increase the carbon flux into flavonoid metabolism. *CgCHI* produces an enzyme that directly catalyzes the transformation of chalcone into the flavanone naringin. Meanwhile, metabolite analysis of transiently overexpressed tobacco and stably overexpressed tomato lines revealed an increase in many flavonoid glycosides and flavonoid acyl glycosides, suggesting that CgMYB1 activates flavonoid biosynthesis pathways in pummelo. Therefore, CgMYB1 may have positive regulatory effects on the phenylpropane pathway in pummelo. Furthermore, many studies have shown that bHLH, MYB, and WD40 can form a MYB-bHLH-WDR (MBW) complex, which co-regulates the phenylpropane metabolic pathway in plants [47–49]. CgMYB1 may also be affected by environmental factors (e.g., light) that co-regulate the flavonoid metabolic pathway in citrus, but this needs further exploration and verification [50].

The study findings not only extend our understanding of the biosynthesis and regulation of phenylpropanoid metabolism in horticultural plants, particularly the metabolic pathways for flavonoid glycoside derivatives in pummelo, but also contribute to efforts to improve nutritional value in pummelo cultivars. Meanwhile, the metabolite correlation data obtained in this study serve as a valuable resource for further investigation of the biosynthetic and regulatory pathways of citrus metabolites.

## Materials and methods

### Population materials

In this study, a total of 189 citrus germplasm resources of different types were collected, mainly including 154 pummelos, as well as 15 madarin, 15 oranges, one lemon, one citron, one papeda, and two *Atalantia buxifolia* (Table S1, see online supplementary material), which representing the mini-core collection of Chinese citrus varieties and various geographical origins, consumption type, and improvement status. These citrus accessions were chosen based on their place of origin and domestication degree. We used the fruit at the commercial mature stage for metabolomic analysis (two biological replicates). For the reasonableness of sampling, we pick five to ten mature fruits around the crown of a tree from three random positions per tree as biological replicate. All the samples were cleaned by sterile deionized water and separate the juice sac tissue immediately. Then, we used liquid nitrogen to freeze samples and stored them at low temperature (−80°C).

### Metabolome analysis

All samples were ground into powder with a zirconia bead (1 min at 30 Hz) by using a mixer mill (MM400, Retsch, Arzberg, Germany). Each powder sample (0.1 g) was immersed in 70% aqueous methanol and pure methanol (1.0 mL) at 4°C for eight hours to extract water and lipid-soluble metabolites, respectively. After centrifugation (10 000 *g* for 10 min) and sedimentation, we use a filter membrane (SCAA-104, 0.22 mm pore size; ANPEL, Shanghai, China, http://www.anpel.com.cn/) to filter the supernatant, and the resulting liquid is used for subsequent metabolic analysis. A multiple reaction monitoring method based on a triple quadrupole-linear ion trap mass spectrometer (API 4000, AB Sciex, Framingham, USA) was used for quantitative analysis of metabolites, and accurate-mass time-of-flight mass spectrometry (6520, Agilent, Santa Clara, California, USA) was used to collect high-resolution data for metabolic signal annotation. The analytical conditions of liquid chromatography were as follows: column, shim-pack VP-ODS C18 (pore size 5.0 μm, length 2 × 150 mm); mobile phases, water (0.04% acetic acid) and acetonitrile (0.04% acetic acid); elution gradient, 0 min 100:0 V/V, 20.0 min 5:95 V/V, 22.0 min 5:95 V/V, 22.1 min 95:5 V/V, 28.0 min 95:5 V/V; flow rate, 0.25 ml min^−1^; temperature, 40°C; injection volume, 2 μl [24]. Lidocaine (0.1 mg l^−1^, Inernal standard) was used to normalize the relative signal intensity of metabolites, and the relative intensity was converted using log_2_. The intensity matrix of 308 runs (154 accessions and two replicate) was generated for the pummelos (Table S3, see online supplementary material).

### Metabolite-based genome-wide association study (mGWAS)

The raw SNP dataset of pummelo accessions can download from the Citrus Pan-genome to Breeding Database (CPBD, http://citrus.hzau.edu.cn/) or NCBI. Minor allele frequency (≥5%) and missing rate (≤10%) were used to filter SNP dataset (*n* = 154 accessions). After filtering, the remaining SNPs were used to execute mGWAS. The GWAS was carried out using linear mixed models as implemented in FaST-LMM [51] and EMMAX [52]. The threshold of *P*-value was calculated by effective number of independent SNPs.

In FaST-LMM, random effects and fixed effects were needed. We used GCTA [53] to compute the first ten principal components and use it as fixed effects. In EMMAX [52], the BN matrix was used to control for population structure. Significant SNPs were merged if within 1000 kb and with a pairwise linkage disequilibrium (*r*^2^) > 0.1. The remaining SNPs were called lead SNPs.

### Construction of a metabolite-SNP-gene network

Associations between metabolites and lead SNPs were explored using mGWAS. Correlations among metabolites were obtained using data on metabolite content in different citrus accessions. The position of each lead SNP on its chromosome was used to find associated genes. Cytoscape (v3.7.1) utilized the above data to construct metabolite-SNP-gene networks.

### Heterologous expression and protein purification

Proteins from candidate genes (*CgMaTs*) were obtained by heterologous expression in *E. coli* [9]. ClonExpress MultiS One Step Cloning Kit (Vazyme) was used to recombine the full cDNA of gene into a modified pGEX-6p-1 expression vector (Novagen), and then expressed in *E. coli* BL21 (DE3) (Weidi Biotechnology). LB liquid medium containing ampicillin was used to culture single colonies. When OD_600nm_ was achieved 0.6–0.8, we added IPTG (with a final concentration of 0.1 mM) in medium and continued to cultivate for 16 h at 20°C. High-pressure homogenizer was used to disrupte cells (6000 *g*/10 min) and cellular debris were removed via centrifugation (14 000 *g*, 1 h). Glutathione sepharose 4B agarose (GE Healthcare) was used to purify target proteins and the proteins were confirmed by using SDS-PAGE. All purified proteins were stored at −80°C for further analysis. Assay conditions for recombinate enzymes.

The 20 μl reaction mixture [38] was configured for acyltransferases CgMaTs, which contains purified enzyme (500 ng), flavonoid substrate (50 μM), malonyl-CoA (50 μM), EDTA (1 mM), and Na-Pi buffer (100 mM and pH = 7.0). After the addition of the enzyme, the reactions were incubated at 37°C for 30 min. Ice-cold methanol (20 μl) were added to terminate reactions and used LC–MS to analyse the products. Both of experimental and technical replicates were performed for each reaction. All substrates (flavonoids, malonyl-CoA and *p*-coumaryl-CoA) were purchased form BioBioPha (http://www.biobiopha.com/), ChemFaces (http://www.chemfaces.cn/), and YuanYe (http://www.shyuanye.com/index.html).

### Transient expression in *Nicotiana benthamiana*

Gateway recombination (Invitrogen) was used for constructing gene expression vector (Table S15, see online supplementary material). The full coding sequences of gene was cloned into entry vector (pDONR207), and then into the destination vector (pEAQ-HT-DEST2). Next, the vectors were transformed into Agrobacterium strain (EHA105). LB liquid medium (5 mL) containing kanamycin (50 μg/mL) were used to cultivate the positive colonies in a shaker (28°C and 200 rpm). When the OD600 reached 0.5–0.8, we stopped shaking the medium and performed further analysis. We used a buffer (10 mM MgCl2, 10 mM MES pH 5.6, and 250 μM acetosyringone) to resuspend cells and adjusted the OD600 to 1.0. We grew cell suspensions at room temperature and under light conditions for 2 h. Then, we infiltrate the suspensions into leaves (4-week-old) of *N. benthamiana*. As a negative control, green fluorescent protein (GFP) containing the pEAQ-HT expression construct was used. In a growth chamber (25°C), the infiltrated plants were grown with a photoperiod of 16/8 h (light/dark). After 1 day, the leaves were collected and were used for LC–MS analysis with the same method as above.

### Statistical analysis

Figure legends contain statistical test(s) method, sample size, and other details. Microsoft Excel (2019) was used to statistical analysis and unpaired two-tailed Student’s *t*-tests was used to compare two data sets. Results are presented as means ± SDs. Significance is displayed by symbols (*P < 0.05, **P < 0.01, ***P < 0.001).

## Supplementary Material

Web_Material_uhad267
