# Supplementary material for: A metabolomics study in citrus provides insight into bioactive phenylpropanoid metabolism
Source: Hortic Res. 2023 Dec 19;11(1):uhad267. doi: 10.1093/hr/uhad267 (PMC10831325; doi:10.1093/hr/uhad267)
Supplement: Web_Material_uhad267 [file web_material_uhad267.zip › Supplementary figures and tables.docx]

# Supplementary figures and tables


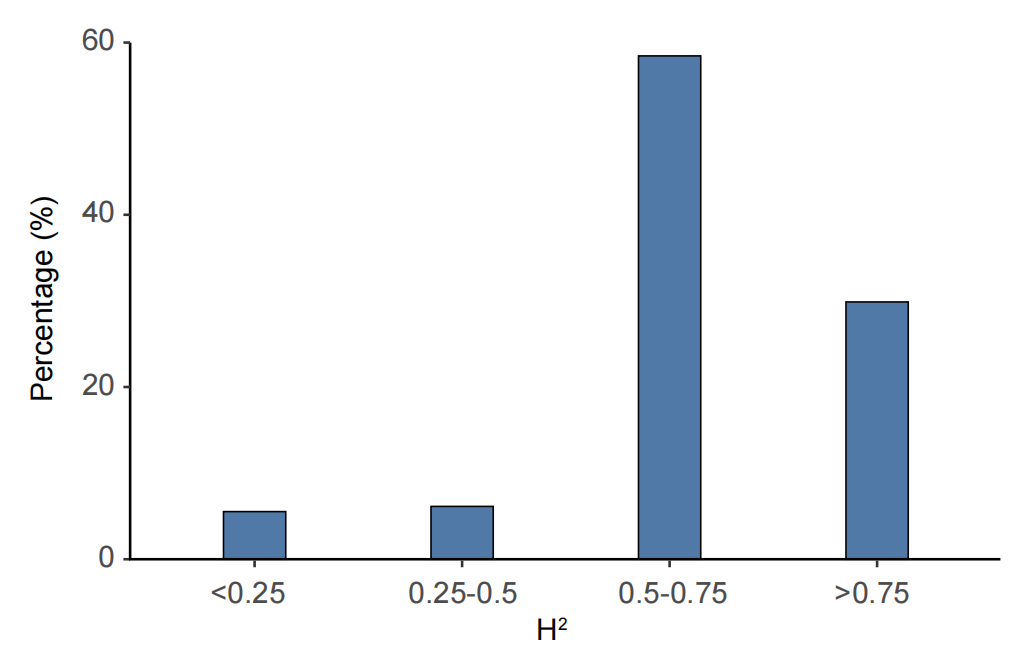


## Figure S1. The distribution of broad-sense heritability (H2) values for metabolites sampled from 154 pummelo accessions.


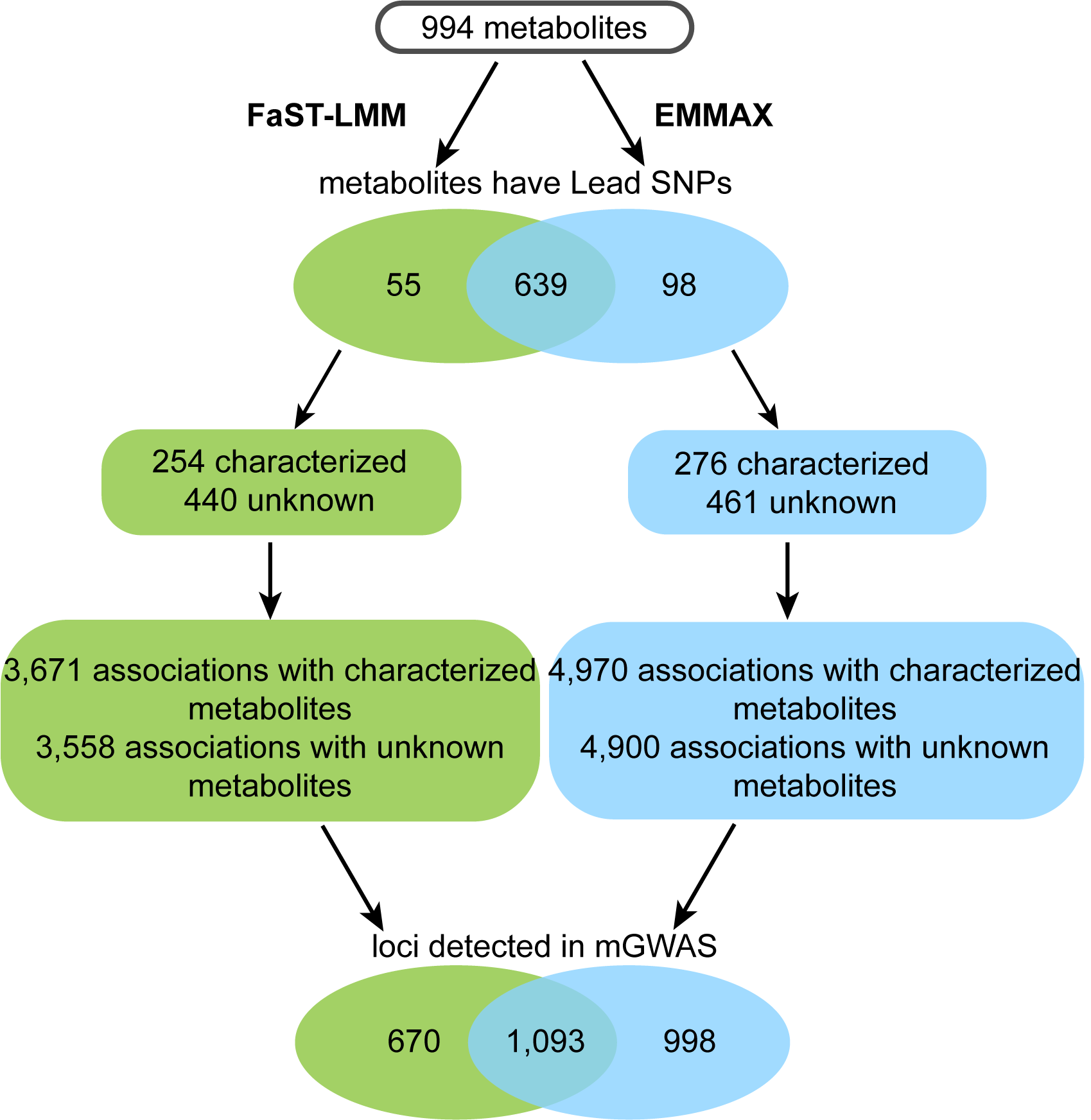


## Figure S2. Schematic overview of the mGWAS for 994 qualitative metabolic traits.


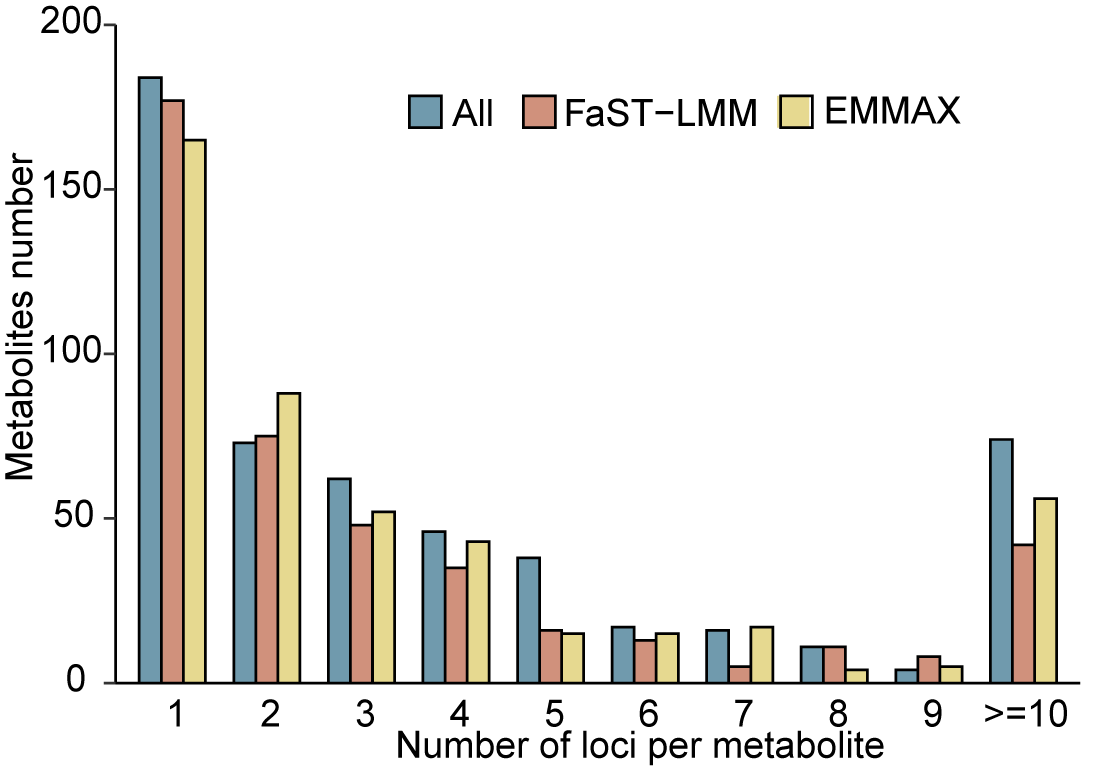


## Figure S3. The number of loci associated with each metabolite using different GWAS methods.


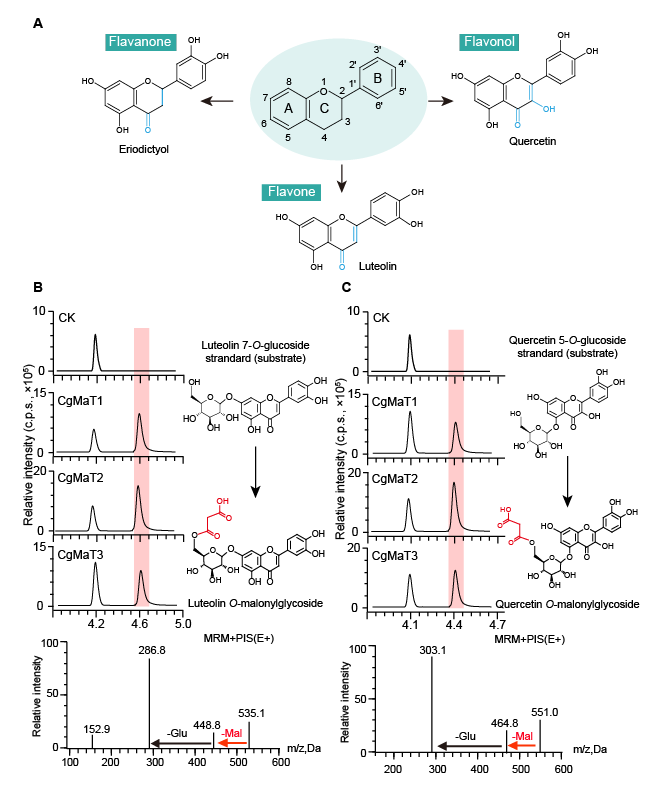


## Figure S4. Enzymatic assays of recombinant flavonoid CgMaT proteins.

(A) Basic chemical structure and classification of flavonoids. The basic structure consists of A, B, and C rings with group positions numbered. The structure of three simple flavonoids, flavanone (eriodictyol), flavone (luteolin), and flavonol (quercetin), discussed in this study is shown.

(B and C) HPLC chromatograms of the reaction of each CgMaT with malonyl-CoA and luteolin 7-*O*-glucoside (B) and quercetin 5-*O*-glucoside (C) as substrate. The MS spectrums and chemical structure of the products luteolin *O*-malonylglycoside (B) and quercetin *O*-malonylglycoside (C) generated from the enzymatic assays.


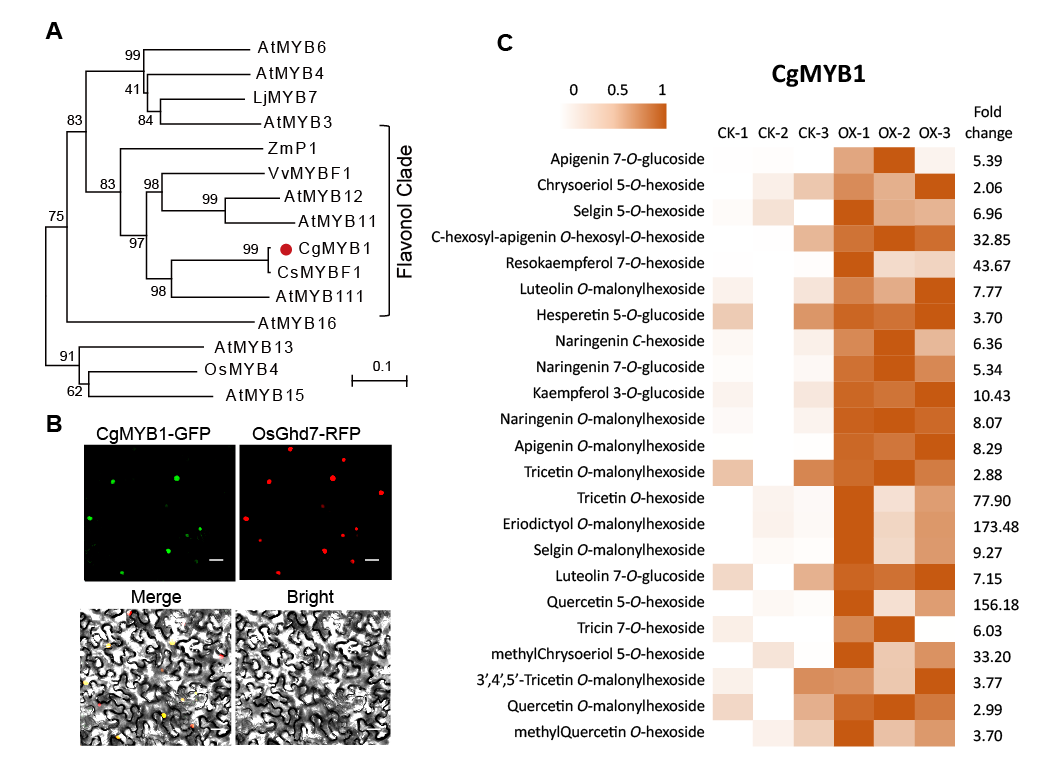


## Figure S5. Validation that CgMYB1 confers transcription activation.

(A) Phylogenetic analysis of select plant MYB proteins. The scale bar represents 0.1 substitutions per site.

(B) Subcellular localization of CgMYB1 in tobacco. CgMYB1-GFP and OsGhd7-RFP were co-transformed into tobacco leaves. OsGhd7-RFP was used as a nuclear marker.

(C) Heat map of metabolite accumulation associated with transient expression of CgMYB1 in tobacco.

## Table S1 A list of the 189 citrus varieties included in the study.

## Table S2 Scheduled MRM transitions for the widely-targeted metabolite analysis of citrus fruits.

## Table S3 Data matrix (log2-transformed) of 994 metabolites identified in citrus fruits.

## Table S4 Loadings (correlation coefficients between the original variables and the principal components) for the three PCs (PC1, PC2, and PC3) for various citrus species.

## Table S5 Metabolic profiles and comparative analysis of pummelo, sweet orange, and mandarins.

## Table S6 Estimated effective number of SNPs and significance thresholds in the citrus population.

## Table S7 The list of lead SNPs detected via mGWAS as carried out using FaST-LMM.

## Table S8 The list of lead SNPs detected via mGWAS as carried out using EMMAX.

## Table S9 The list of all lead SNPs detected via mGWAS.

## Table S10 The list of all loci detected using mGWAS.

## Table S11 The full list of significant associations from mGWAS.

## Table S12 The full list of highly significant associations from mGWAS.

## Table S13 Correlation matrix between varieties.

## Table S14 Data used to construct coumarin and flavonoid subnetworks.

## Table S15 Primers used in this study.
